# CD127^+^ Natural Killer Cells Represent a Distinct, Interleukin-15-Independent and Thymus-Independent Subset in Mice

**DOI:** 10.3390/ijms27062667

**Published:** 2026-03-14

**Authors:** Yuna Kim, Seon-Yeong Hwang, Young-Jin Kwon, Ji-Eun Kim, Lata Rajbongshi, Su-Rin Lee, Seongwon Joo, Seongheum Park, Sae-Ock Oh, Byoung-Soo Kim, Dongjun Lee, Sik Yoon

**Affiliations:** 1 School of Medicine, Pusan National University, Yangsan 50612, Republic of Korea; yuna00kim@pusan.ac.kr (Y.K.); jsw621@naver.com (S.J.); tkdrh30@naver.com (S.P.); 2 Department of Anatomy, School of Medicine, Pusan National University, Yangsan 50612, Republic of Korea; anatomy2017@pusan.ac.kr (S.-Y.H.); kyj5296@hanmail.net (Y.-J.K.); ji.eun@pusan.ac.kr (J.-E.K.); latapharm@gmail.com (L.R.); su020512@naver.com (S.-R.L.); hedgehog@pusan.ac.kr (S.-O.O.); 3 School of Biomedical Convergence Engineering, Pusan National University, Yangsan 50612, Republic of Korea; bskim7@pusan.ac.kr; 4 Department of Convergence Medicine, Pusan National University College of Medicine, Yangsan 50612, Republic of Korea; lee.dongjun@pusan.ac.kr; 5 Research Institute for Convergence of Biomedical Science and Technology, Pusan National University Yangsan Hospital, Yangsan 50612, Republic of Korea

**Keywords:** natural killer (NK) cell, CD127 (IL-7Rα), innate lymphoid cell (ILC), IL-7, IL-15, mouse model

## Abstract

Natural killer (NK) cells, key effectors of innate immunity, are classically categorized into CD56^dim^ and CD56^bright^ subsets in humans. While murine NK cell heterogeneity has become increasingly recognized, the classification of mature NK cell subsets remains incompletely defined. Here, we comprehensively characterized CD127^+^ NK cells in mice and identified them as a distinct, mature subset, developing independently of the thymus and interleukin (IL)-15 signaling. Flow cytometric analyses revealed that CD127^+^ NK cells are broadly distributed across lymphoid and non-lymphoid tissues—including in C57BL/6 wild-type and athymic Foxn1^−/−^ mice—and exhibit a surface phenotype distinct from CD127^−^ NK and thymus-derived CD127^+^ NK cells. Functional assays demonstrated that CD127^+^ NK cells produce interferon-γ and exert cytotoxic activity, despite expressing markers typically associated with immature NK cells. CD127^+^ NK cells were absent in IL-7Rα^−/−^ mice but present in IL-15^−/−^ and IL-15Rα^−/−^ mice, indicating a selective dependence on IL-7 signaling. IL-7 promoted their proliferation and activation both in vitro and in vivo. These findings revise current models of NK cell development by identifying a novel, IL-7-responsive, IL-15-independent, thymus-independent, and functionally competent CD127^+^ NK cell subset that is phenotypically distinct from helper-like innate lymphoid cells (ILCs). This study provides a framework for future investigations on NK cell heterogeneity, tissue specialization, and cytokine-mediated regulation.

## 1. Introduction

Natural killer (NK) cells are innate lymphocytes capable of recognizing and eliminating target cells without prior sensitization. Unlike T and B cells, NK cells play critical roles in immune defense against tumors and a wide range of pathogens, including viruses, bacteria, and parasites [1,2,3]. In addition, these cells contribute to immune regulation in contexts such as autoimmunity and inflammation [2,4]. NK cells develop independently of antigen receptor gene rearrangement, and their activity is governed by a balance of activating and inhibitory signals mediated through receptors that interact with MHC class I molecules and related ligands [5,6].

In mice, NK cell development occurs primarily in the bone marrow and proceeds through multiple phenotypically and functionally distinct stages [4,7,8,9,10,11,12,13,14]. Hematopoietic stem cells generate common lymphoid progenitors (CLPs), which differentiate into bipotent T/NK progenitors (T/NKPs), NK progenitors (NKPs), and ultimately mature NK cells [9,11,15]. CLPs (Lin^−^c-Kit^low^IL-7Rα^+^Sca-1^low^) can generate B, T, and NK lineages, with their fate shaped by environmental cues [9,16,17,18,19]. Interleukin (IL)-15 is critical for NK cell development and homeostasis, regulating their survival, proliferation, and maturation [20]. The transition from T/NKPs to NKPs is marked by the acquisition of IL-2/IL-15Rβ (CD122), which enables responsiveness to IL-15 [15,17,21]. IL-15 signals through a heterotrimeric receptor composed of IL-15Rα, CD122, and the common γ chain (γc, CD132) [22]. Mice deficient in IL-15, IL-15Rα, or IL-2/IL-15Rβ exhibit impaired NK cell development, while γc deficiency results in broader lymphoid defects and a complete loss of mature NK cells [23,24,25,26,27,28].

As NKPs mature, they acquire surface receptors such as CD94/NKG2 and Ly49, which mediate the recognition of MHC class I molecules [9]. CD127 (IL-7Rα) is downregulated during maturation, whereas CD122 expression is upregulated [12]. In IL-15- or IL-15Rα-deficient mice, NK cell development is arrested at the NKP stage, with few peripheral NK cells detectable [28,29,30,31]. However, the phenotype and function of CD127^+^ NK cells that arise through alternative developmental pathways remain incompletely defined.

Murine NK cell development frequently occurs in five stages. Stage I NKPs express CD122, while lacking NK1.1 and DX5 (CD49b, integrin α2) [8,32,33]. By stage II, NK1.1 expression begins, along with the emergence of integrin αv and CD94/NKG2 [8,32,33,34]. Ly49 receptor expression is initiated at stage III, followed by DX5 expression and active proliferation at stage IV [34,35,36]. Integrin αv is typically lost as NK cells acquire Mac-1 (CD11b/CD18, integrin αMβ2) and CD43 at stage V, marking terminal maturation and acquisition of cytotoxic function [8,34,35,36].

Although these developmental stages provide a useful framework, murine NK cell maturation is highly complex and is regulated by transcription factors such as T-bet, Eomes, Zeb2, and Bach2, as well as by transcriptional and epigenetic rewiring [4,9,37,38,39,40,41]. Subset distinctions, such as CD27^+^CD11b^+^ versus CD27^−^CD11b^+^ murine NK cells, remain under investigation, particularly regarding their roles in tissue residency and memory-like responses [42,43].

Two major NK subsets are recognized in humans: CD56^dim^ NK cells (cytotoxic) and CD56^bright^ NK cells (cytokine-producing), which differ in surface marker expression, function, and tissue distribution [44,45,46]. Because mice lack CD56, murine NK cell subsets are classified using alternative markers, particularly CD27 and Mac-1. Based on this system, CD27^high^Mac-1^high^ NK cells have been proposed to developmentally resemble human CD56^bright^ NK cells, although they exhibit increased cytotoxicity [47,48,49,50]. In contrast, CD27^low/−^Mac-1^high^ cells are considered more differentiated and possibly senescent, sharing some features with human CD56^dim^ NK cells, although they lack a clear one-to-one correspondence [47,48,49,50].

Additionally, CD127^+^ NK cell populations have been identified in mice, including a CD27^high^Mac-1^low^CD127^+^ subset that may represent an early developmental precursor [47] and thymic emigrants that partially resemble human CD56^bright^ NK cells [51]. Furthermore, the discovery of innate lymphoid cells (ILCs) has added complexity to this classification, as both mature NK cells and helper-like ILC1s can express NK1.1, CD122, and CD127 [52]. Distinguishing peripheral CD127^+^ NK populations from ILC1s requires the assessment of Eomes, CD49b (DX5), and CD11b (Mac-1) expression [52,53,54,55,56]. Despite these efforts so far, the classification of murine NK subsets—particularly the lineage and tissue distribution of peripheral CD127^+^ NK cells—remains poorly resolved.

In this study, CD127^+^ and CD127^−^ NK cell subsets were comprehensively characterized across multiple mouse tissues. We identified a mature, IL-15-independent, IL-7-responsive, and largely thymus-independent CD127^+^ NK cell population that is distinct from previously described thymus-derived NK cells [51]. These findings provide new insights into NK cell heterogeneity and ontogeny in mice.

## 2. Results

### 2.1. Tissue Distribution and Age-Related Stability of CD127^+^ NK Cells in Mice

**Figure 1:**
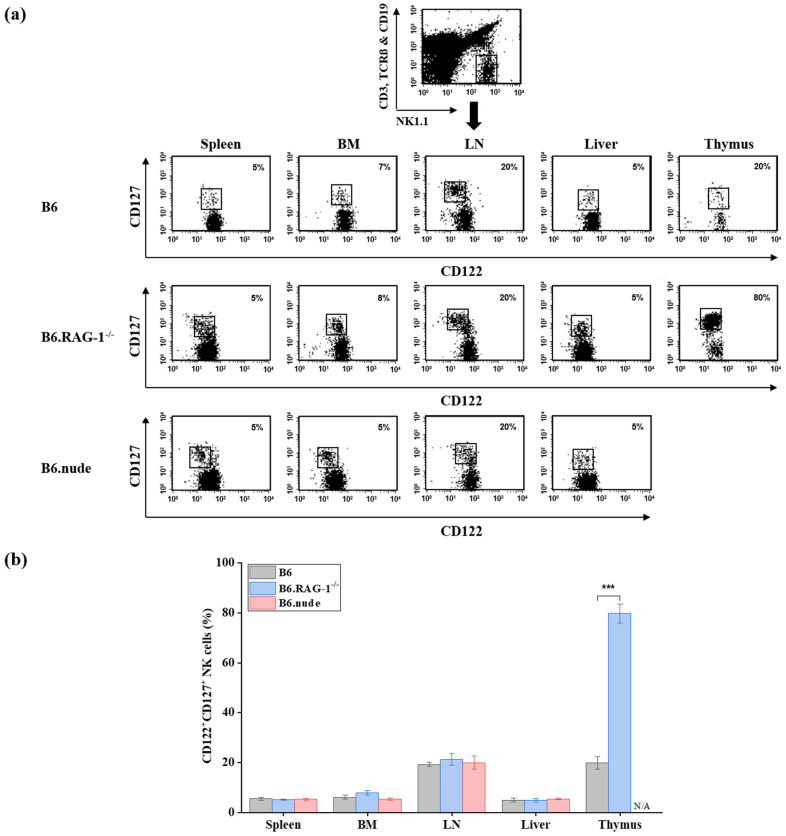
Distribution of CD127^+^ NK cells across multiple murine tissues. (**a**) Representative flow cytometric analysis of CD122 and CD127 expression on gated NK cells (NK1.1^+^CD3^−^TCRβ^−^CD19^−^) from C57BL/6 (B6), B6.RAG-1^−/−^, and athymic nude Foxn1^−/−^ (B6.nude) mice (*n* = 5 per group). Percentages in the upper right quadrant indicate the frequency of CD122^+^CD127^+^ NK cells (boxed) within the NK cell gate. (**b**) Quantitative analysis of CD122^+^CD127^+^ NK cell frequencies in the spleen, bone marrow (BM), lymph nodes (LN), liver, and thymus. Data are presented as mean ± SD. Statistical comparisons with B6 controls in the spleen, BM, LN, and liver were performed using one-way ANOVA followed by Dunnett’s post hoc test. Thymic comparisons were analyzed using an unpaired two-tailed Student’s *t*-test. Statistical significance is indicated as *** p < 0.001. N/A, not applicable; thymic data for B6.nude mice are not applicable due to thymic aplasia. NK—natural killer.

To characterize CD127 expression on murine NK cells, we performed flow cytometric analysis of gated NK cells (NK1.1^+^CD122^+^CD3^−^TCRβ^−^CD19^−^) isolated from mononuclear cells of the spleen, bone marrow, lymph nodes, liver, and thymus of C57BL/6 (B6) mice. A distinct subset of CD127^+^ NK cells was consistently detected across all examined tissues, with moderate inter-individual variability (Figure 1). The frequencies of CD127^+^ NK cells in the spleen, bone marrow, lymph nodes, and liver were comparable to those previously reported [51]. In contrast, only approximately 20% of thymic NK cells expressed CD127, which is substantially lower than the ~85% reported in earlier studies (Figure 1), prompting further investigation into thymic contribution.

In B6.RAG-1^−/−^ mice, which lack mature T and B cells, the frequency of CD127^+^ NK cells in the thymus increased markedly to approximately 80%, representing an approximately four-fold increase compared with wild-type controls (*p* < 0.001). In peripheral tissues (spleen, BM, LN, and liver), CD127^+^ NK cell frequencies exhibited only minor, non-significant differences relative to wild-type mice (0.9- to 1.3-fold changes; Figure 1).

To further assess thymic dependence, we analyzed athymic Foxn1^−/−^ (B6.nude) mice. In these mice, CD127^+^ NK cell frequencies within the NK cell gate were comparable to wild-type controls in the spleen, bone marrow, lymph nodes, and liver (fold changes: 1.0, 0.9, 1.0, and 1.1, respectively). As expected, thymic analysis was not applicable due to thymic aplasia.

Collectively, these findings indicate that a substantial population of CD127^+^ NK cells exists independently of thymic development. Nevertheless, the marked enrichment observed in RAG-1^−/−^ mice suggests that thymus-derived or lymphopenia-associated mechanisms may facilitate CD127^+^ NK cell expansion under specific conditions.

**Figure 2:**
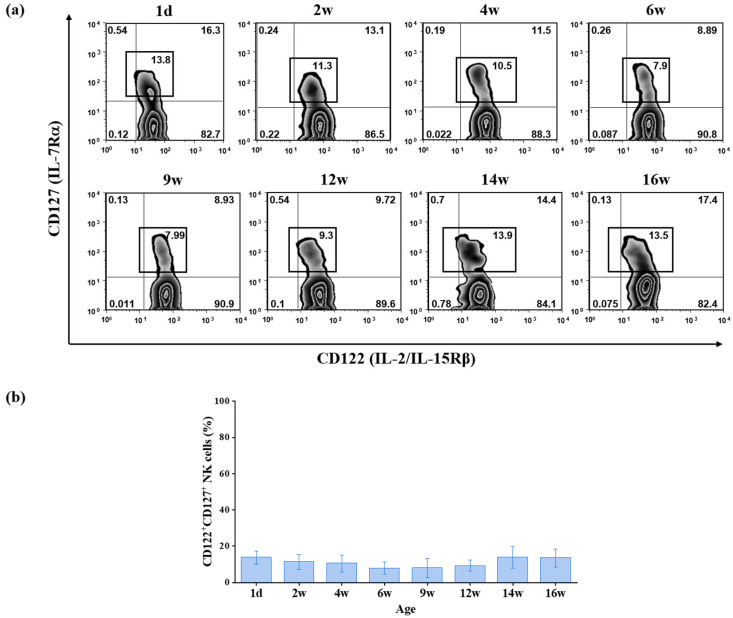
Frequencies of CD127^+^ and CD127^−^ NK cells across postnatal development in C57BL/6 mice. (**a**) Representative flow cytometric analysis of CD122 and CD127 expression on gated splenic NK cells (NK1.1^+^CD3^−^TCRβ^−^CD19^−^) from C57BL/6 (B6) mice at different ages (1 day; 2, 4, 6, 9, 12, 14, and 16 weeks). The percentages shown in the boxed region indicate the frequency of CD122^+^CD127^+^ NK cells within the NK cell gate. (**b**) Quantitative analysis of CD122^+^CD127^+^ NK cell frequencies. Data are presented as mean ± SD (*n* = 5 mice per age group). Statistical comparisons versus day 1 controls were performed using one-way ANOVA followed by Dunnett’s multiple comparisons test. No statistically significant differences were observed (*p* > 0.05). Age is indicated in days (d) or weeks (w). IL—interleukin.

To determine whether CD127^+^ NK cell distribution changes during postnatal development, we assessed the frequencies of CD127^+^ NK cells in the spleens of neonatal (day 1), juvenile, and adult C57BL/6 mice. Across all age groups examined, the proportion of CD127^+^ cells within the NK1.1^+^CD122^+^CD3^−^TCRβ^−^CD19^−^ compartment remained relatively stable (Figure 2). One-way ANOVA of cumulative data confirmed no statistically significant changes in subset frequency from birth to adulthood (*p* > 0.05; Figure 2b). Thus, CD127^+^ NK cell frequency appears to be maintained at a relatively constant level throughout postnatal development.

### 2.2. Phenotypic Characterization of CD127^+^ NK Cells in Mice

**Figure 3:**
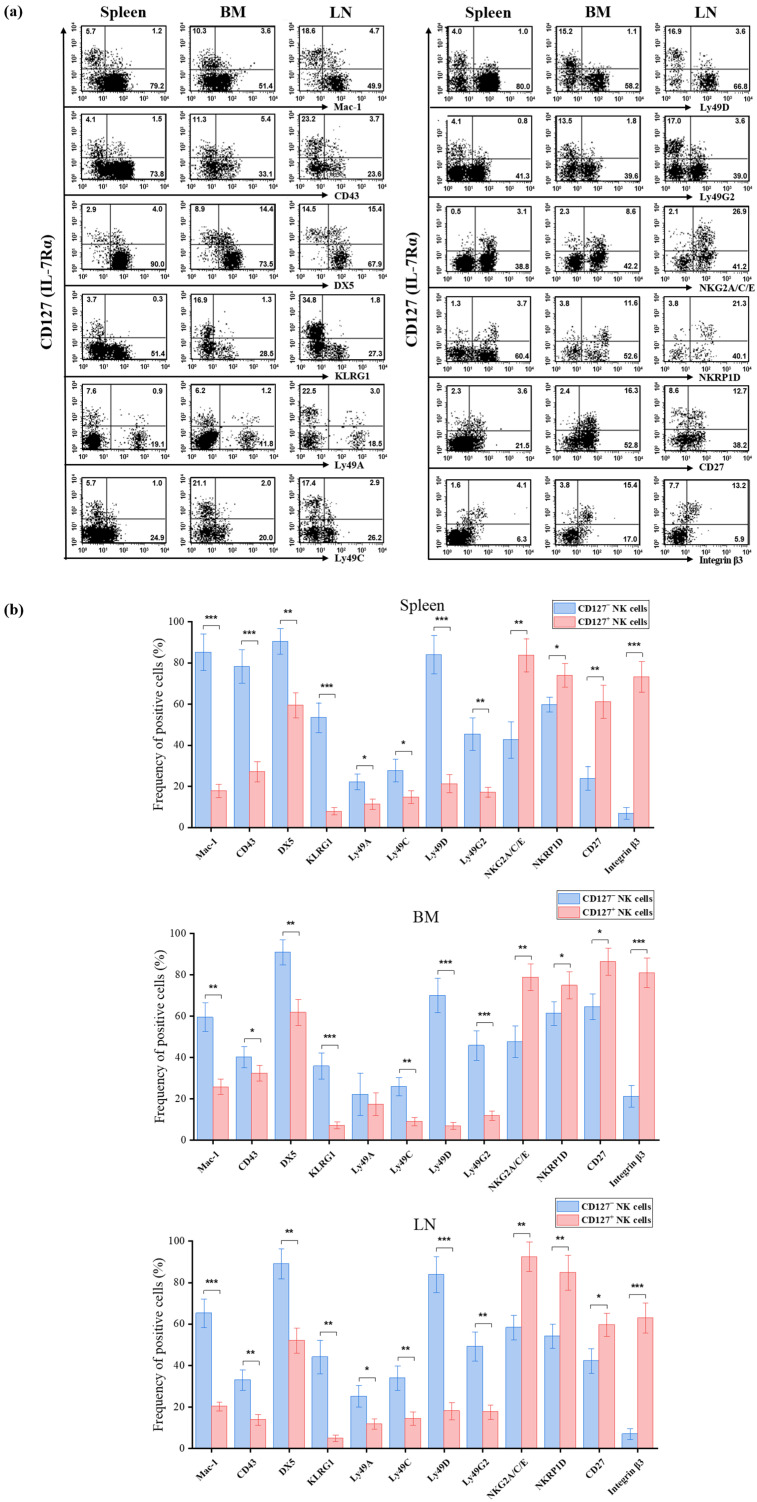
Phenotypic profile of murine CD127^+^ NK cells. (**a**) Representative flow cytometric analysis of CD127 expression and multiple NK cell surface markers on gated NK cells (NK1.1^+^CD3^−^TCRβ^−^CD19^−^) from the spleen, bone marrow (BM), and lymph nodes (LN) of C57BL/6 mice (*n* = 3). Surface markers analyzed include Mac-1, CD43, DX5, KLRG1, Ly49A, Ly49C, Ly49D, Ly49G2, NKG2A/C/E, NKRP1D, CD27, and integrin β3. Numbers in the quadrants indicate the percentage of cells within each region. (**b**) Quantitative summary of marker expression frequencies on CD127^−^ and CD127^+^ NK cells. Data are presented as mean ± SD. Statistical comparisons between CD127^+^ and CD127^−^ NK cells within each tissue were performed using an unpaired two-tailed Student’s *t*-test. Statistical significance is indicated as * *p* < 0.05, ** *p* < 0.01, and *** *p* < 0.001.

To define the phenotypic characteristics of CD127^+^ NK cells, we analyzed splenic, bone marrow, and lymph node NK cells from C57BL/6 mice using a comprehensive panel of monoclonal antibodies. CD127^+^ NK cells were directly compared with CD127^−^ NK cells isolated from the same organs (Figure 3).

In the spleen, CD127^+^ NK cells exhibited significantly lower expression of maturation-associated markers compared with CD127^−^ NK cells, as evidenced by the frequencies of Mac-1 (17.8 ± 3.2% vs. 85.1 ± 8.9%, *p* < 0.001), CD43 (27.1 ± 4.8% vs. 78.2 ± 8.2%, *p* <0.001), DX5 (59.4 ± 6.2% vs. 90.4 ± 6.3%, *p* < 0.01), and KLRG1 (7.9 ± 1.8% vs. 53.4 ± 7.2%, *p* < 0.001) (Figure 3a,b). Similarly, multiple Ly49 receptors (Ly49A, Ly49C, Ly49D, and Ly49G2) were expressed at significantly lower frequencies in CD127^+^ NK cells (all *p* < 0.05) (Figure 3a,b). In contrast, CD127^+^ NK cells displayed significantly higher expression of NKG2A/C/E^+^ cells (83.6 ± 8.1% vs. 42.6 ± 8.9%, *p* < 0.01), NKRP1D^+^ cells (73.9 ± 5.7% vs. 59.7 ± 3.6%, *p* < 0.05), CD27^+^ cells (61.1 ± 7.9% vs. 23.8 ± 5.8%, *p* < 0.01) and integrin β3^+^ cells (73.2 ± 7.4% vs. 6.9 ± 2.9%, *p* < 0.001), compared with CD127^−^ NK cells (Figure 3a,b).

This pattern was consistently observed across bone marrow and lymph nodes. In the bone marrow, CD127^+^ NK cells showed reduced expression of Mac-1, CD43, DX5, and KLRG1, as well as decreased frequencies of Ly49C, Ly49D, and Ly49G2 (all *p* < 0.05–0.001), whereas Ly49A showed no significant difference (Figure 3b). Conversely, NKG2A/C/E, NKRP1D, CD27, and integrin β3 were significantly enriched in CD127^+^ NK cells (all *p* < 0.05–0.001) (Figure 3a,b).

A similar phenotypic pattern was observed in lymph nodes. CD127^+^ NK cells exhibited reduced expression of maturation markers and Ly49 receptors but increased frequencies of NKG2A/C/E^+^, NKRP1D^+^, CD27^+^, and integrin β3^+^ cells relative to CD127^−^ NK cells (all *p* < 0.05–0.001) (Figure 3a,b).

Collectively, these data indicate that murine CD127^+^ NK cells exhibit a phenotypic profile consistent with a less mature or developmentally distinct subset. Crucially, while ILC1s are typically characterized as CD127^+^DX5^−^Mac-1^−^, the population identified here maintains reduced but significant expression of Mac-1 and DX5 across multiple tissues, supporting their classification within the NK cell lineage rather than as ILC1s [52,53,54,55,56].

**Figure 4:**
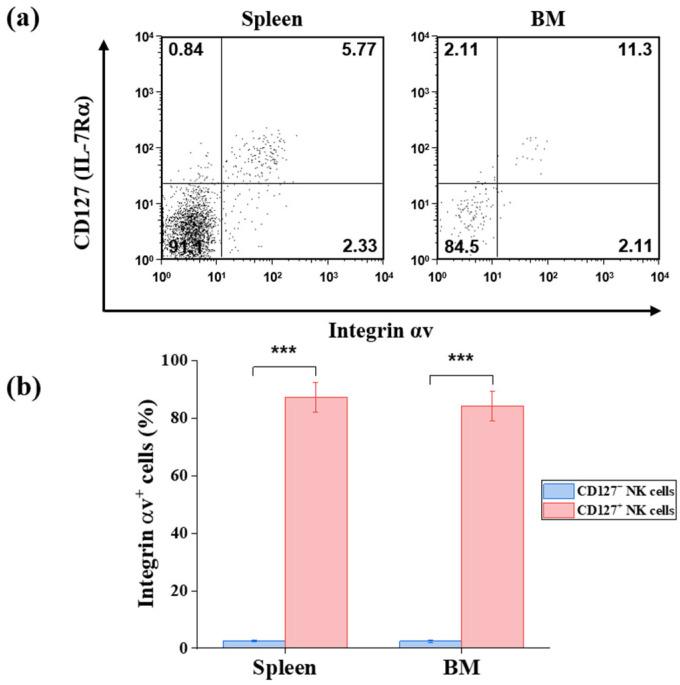
Differential expression of integrin αv on murine CD127^+^ and CD127^−^ NK cell subsets (*n* = 3). (**a**) Representative flow cytometric analysis of integrin αv expression on gated NK cells (NK1.1^+^CD3^−^TCRβ^−^CD19^−^) from the spleen and bone marrow (BM) of C57BL/6 mice. Numbers in the quadrant indicate the percentage of cells within each region. (**b**) Quantitative analysis of integrin αv expression frequencies on CD127^+^ and CD127^−^ NK cells. Data are presented as mean ± SD. Statistical comparisons between CD127^+^ and CD127^−^ NK cells were performed using an unpaired two-tailed Student’s *t*-test. Statistical significance is indicated as *** *p* < 0.001.

Additionally, CD127^+^ NK cells exhibited markedly elevated expression of integrin αv compared with CD127^−^ NK cells. In the spleen, 87.3 ± 5.2% of CD127^+^ NK cells expressed integrin αv, whereas only 2.5 ± 0.4% of CD127^−^ NK cells were positive (*p* < 0.001) (Figure 4). A similarly pronounced difference was observed in the bone marrow (84.3 ± 2.7% vs. 2.4 ± 0.5%, *p* < 0.001) (Figure 4). These findings identify integrin αv as a distinguishing surface marker highly enriched in the CD127^+^ NK cell subset.

### 2.3. Murine CD127^+^ NK Cells Do Not Represent Immature NK Cells at Early Developmental Stages

The reduced expression of maturation-associated markers on CD127^+^ NK cells suggested that this subset might represent an immature NK cell population. To test this possibility, we examined CD127^+^ NK cells in settings characterized by early immune development or NK cell immaturity, including fetal (gestational day 15.5), neonatal (postnatal day 1), and adult NKD mice.

**Figure 5:**
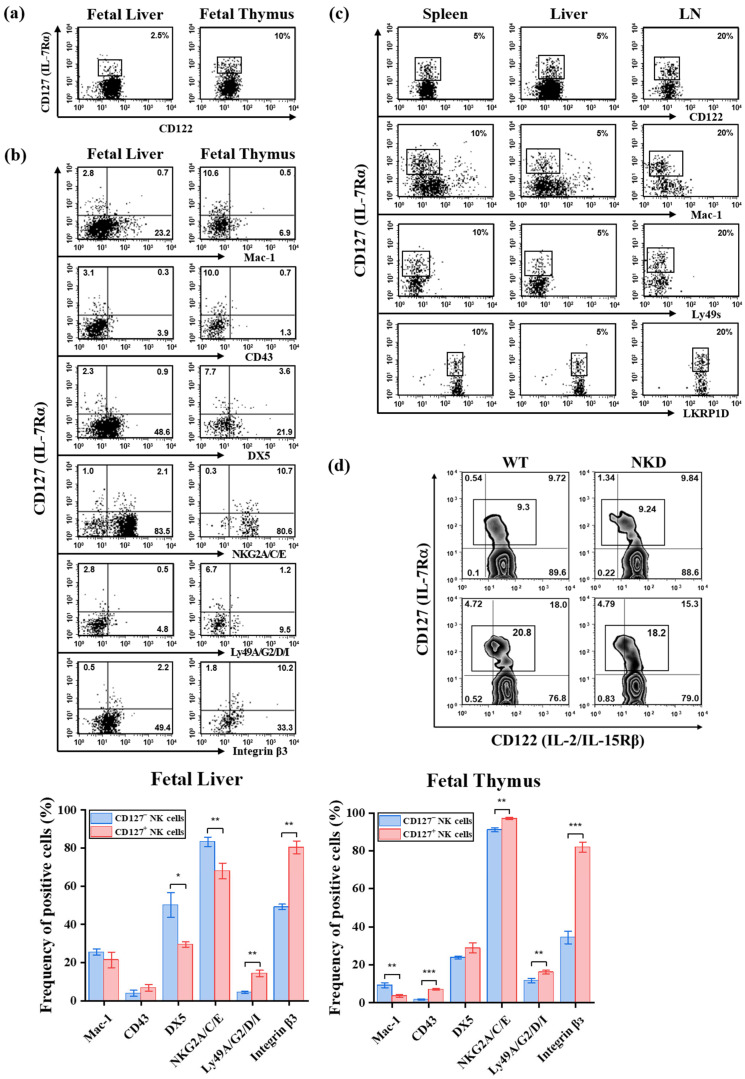
CD127^+^ NK cells are not restricted to immature developmental stages. (**a**) Representative flow cytometric analysis of CD122 and CD127 expression on gated NK cells (NK1.1^+^CD3^−^TCRβ^−^CD19^−^) isolated from the fetal liver and thymus of C57BL/6 mice at gestational day 15.5 (*n* = 3). Boxed regions indicate the frequency of CD127^+^ NK cells. (**b**) Representative phenotypic analysis of CD127^+^ and CD127^−^ NK cells from fetal liver and thymus. Surface markers analyzed include maturation-associated markers (Mac-1, CD43, and DX5), inhibitory and activating receptors (NKG2A/C/E, Ly49A/G2/D/I), and integrin β3. Bar graphs summarize the frequency of positive cells (mean ± SD). Bar graphs show the frequency of positive cells (mean ± SD). (**c**) Representative flow cytometric analysis of CD127 expression and selected NK cell surface markers in splenic, hepatic, and lymph node NK cells from neonatal C57BL/6 mice (postnatal day 1) (*n* = 3). (**d**) CD122 and CD127 expression on splenic and lymph node (LN) NK cells from adult wild-type (WT) and NK cell-deficient (NKD) mice (*n* = 3). Numbers in quadrants or boxed regions indicate the percentage of cells within each gate. Statistical comparisons between CD127^+^ and CD127^−^ subsets were performed using an unpaired two-tailed Student’s *t*-test and are indicated as * *p* < 0.05, ** *p* < 0.01, and *** *p* < 0.001.

Flow cytometric analyses were performed on mononuclear cells isolated from the fetal liver and thymus, neonatal spleen, liver, and lymph nodes, as well as spleen and lymph nodes from adult NKD mice (Figure 5). Surface expression of CD127, CD122, NKG2A/C/E, Ly49 receptors, integrin β3, NKRP1D, and maturation-associated markers (Mac-1, CD43, and DX5) was assessed within the gated NK cell population (NK1.1^+^CD3^−^TCRβ^−^CD19^−^).

The frequency of CD122^+^CD127^+^ NK cells in fetal liver and thymus was statistically comparable to that observed in adult C57BL/6 controls (*p* > 0.05) (Figure 5a). In the fetal liver, CD127^+^NK cells also displayed a phenotypic profile statistically comparable to that observed in adult C57BL/6 mice (*p* > 0.05, Figure 5b). Although modest differences were noted in the frequencies of Mac-1, CD43, DX5, NKG2A/C/E, Ly49A/G2/D/I, and integrin β3 compared with CD127^−^ NK cells, the overall expression pattern of CD127^+^ NK cells did not reflect a uniformly immature phenotype (Figure 5b). Similarly, in the fetal thymus, CD127^+^ NK cells maintained high expression of NKG2A/C/E and integrin β3, whereas maturation-associated markers were predominantly reduced within the CD127^−^ subset mice (Figure 5b).

Analysis of neonatal mice yielded comparable results. The frequency, distribution, and surface phenotype of CD127^+^ NK cells in neonatal spleen, liver, and lymph nodes were statistically comparable to those observed in adult C57BL/6 controls (*p* > 0.05) (Figure 5c). In contrast, CD127^−^ NK cells in fetal and neonatal tissues exhibited significantly lower frequencies of Mac-1, CD43, and DX5 compared to adult counterparts (all *p* < 0.05–0.001), confirming an immature phenotype (Figure 5b,c).

Finally, in NKD mice, the proportion of CD127^+^ NK cells was similar to that observed in wild-type controls (Figure 5d), indicating that CD127^+^ NK cells are not preferentially enriched in conditions associated with impaired NK cell maturation.

Collectively, these findings demonstrate that CD127^+^ NK cells do not represent immature NK cells arising during early developmental stages. Instead, their phenotypic characteristics are maintained across fetal, neonatal, and adult settings, supporting the conclusion that CD127^+^ NK cells constitute a developmentally distinct NK cell subset rather than a transient immature stage.

To further determine whether CD127^+^ NK cells represent functionally immature or unlicensed NK cells, we examined NK cells from β2-microglobulin-deficient (β2m^−/−^) mice. Because β2m is required for MHC class I expression and engagement of inhibitory NK receptors—a critical step in the acquisition of functional competence (“licensing”) [57,58,59,60]—β2m^−/−^ mice provide a well-established model for evaluating NK cell maturation status.

Phenotypic analysis of splenic NK cells revealed that CD127^+^ NK cells from β2m^−/−^ mice exhibited expression profiles comparable to those from wild-type C57BL/6 controls. No significant differences were observed in the frequencies of CD43, CD27, Ly49D, or Ly49G2 expression between CD127^+^ NK cells from β2m^−/−^ and wild-type mice. Similarly, the CD127^−^ NK cell subset displayed no significant differences between β2m^−/−^ and wild-type mice with respect to these markers.

**Figure 6:**
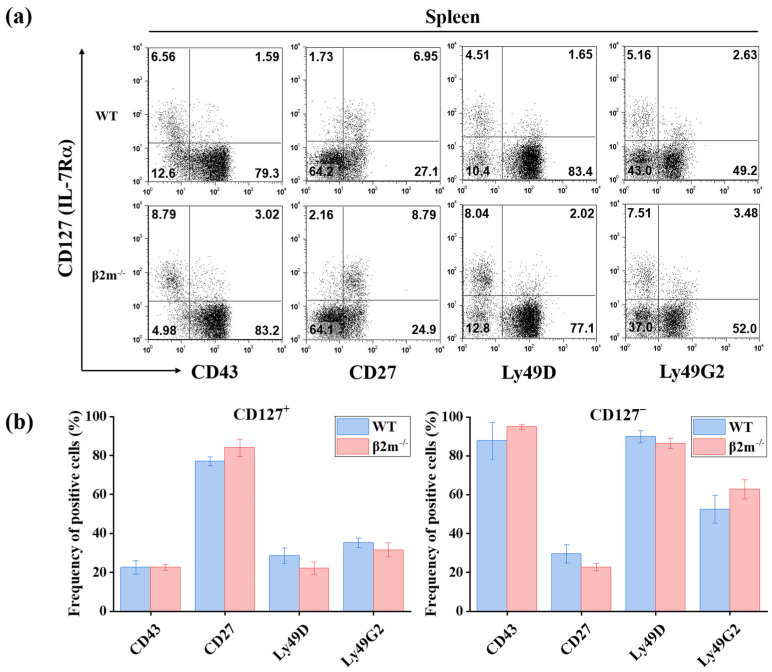
CD127^+^ NK cells are not phenotypically altered in β2m^−/−^ mice (*n* = 3). (**a**) Representative flow cytometric analysis of CD43, CD27, Ly49D, and Ly49G2 expression on gated splenic NK cells (NK1.1^+^CD3^−^TCRβ^−^CD19^−^) from wild-type (WT) and β2m^−/−^ mice. CD127^+^ and CD127^−^ NK cell subsets are shown. Numbers in the quadrants indicate the percentage of cells within each region. (**b**) Quantitative analysis of marker expression frequencies in CD127^+^ and CD127^−^ NK cells from WT and β2m^−/−^ mice. Data are presented as mean ± SD from at least three independent experiments. Statistical comparisons between WT and β2m^−/−^ mice within each subset were performed using an unpaired two-tailed Student’s *t*-test. No statistically significant differences were observed.

These findings indicate that the phenotypic characteristics of CD127^+^ NK cells are maintained independently of MHC class I-mediated licensing signals. Thus, CD127^+^ NK cells do not exhibit features consistent with functional immaturity or defective licensing (Figure 6).

**Figure 7:**
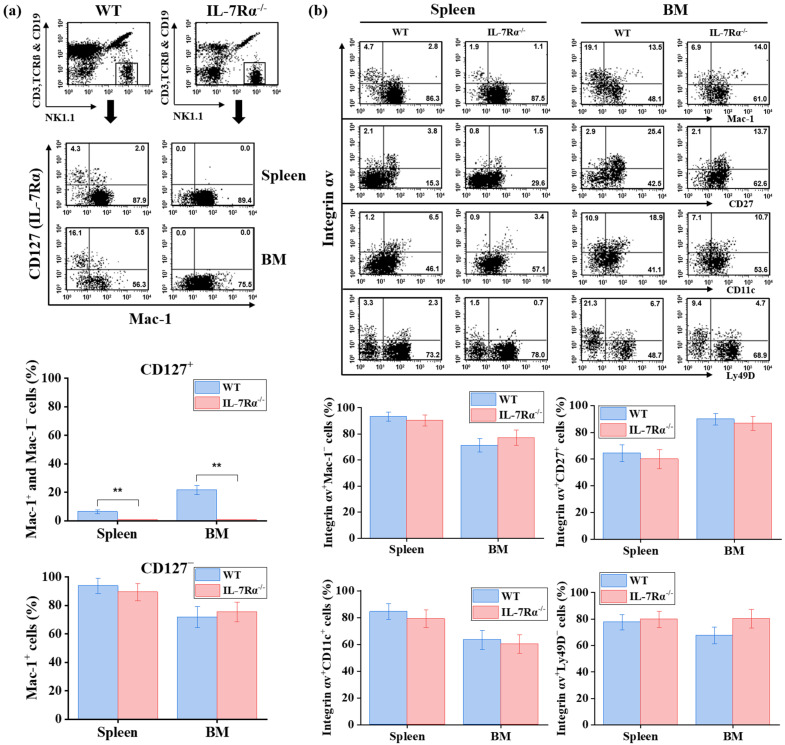
Selective loss of CD127^+^ NK cells in IL-7Rα^−/−^ mice. (**a**) Representative flow cytometric analysis of CD127 and Mac-1 expression on gated NK cells (NK1.1^+^CD3^−^TCRβ^−^CD19^−^) from the spleen and bone marrow (BM) of wild-type (WT) C57BL/6 and IL-7Rα^−/−^ mice (*n* = 3). CD127^+^ NK cells are absent in IL-7Rα^−/−^ mice, whereas CD127^−^ NK cells are preserved. (**b**) Phenotypic analysis of integrin αv, Mac-1, CD27, CD11c, and Ly49D expression on CD127^+^ and CD127^−^ NK cell subsets from the spleen and bone marrow of WT and IL-7Rα^−/−^ mice. Quantitative analysis of marker expression frequencies is shown. Numbers in quadrants indicate the percentage of cells within each gate. Data are presented as mean ± SD. Statistical comparisons between WT and IL-7Rα^−/−^ mice within each subset were performed using an unpaired two-tailed Student’s *t*-test and are indicated as ** *p* < 0.01. No statistically significant differences were observed in the CD127^−^ subset.

To determine whether IL-7Rα signaling is required for the development of CD127^+^ NK cells and to assess whether CD127^+^ NK cells represent a precursor stage of CD127^−^ NK cells, we analyzed NK cell populations in the spleen and bone marrow of IL-7Rα^−/−^ mice. Flow cytometric analysis revealed that CD127^+^ NK cells were completely absent in IL-7Rα^−/−^ mice, whereas CD127^−^ NK cells were preserved (Figure 7a).

Phenotypic analysis demonstrated that CD127^−^ NK cells from IL-7Rα^−/−^ mice exhibited expression levels of Mac-1 comparable to those observed in wild-type controls, indicating that conventional NK cell development was largely intact (Figure 7a).

To exclude the possibility that the apparent absence of CD127^+^ NK cells reflected CD127 downregulation rather than loss of a distinct subset, we further analyzed markers that distinguish CD127^+^ and CD127^−^ NK cells, including integrin αv, Mac-1, CD27, CD11c, and Ly49D (Figure 7b). In IL-7Rα^−/−^ mice, independent surrogate cell populations that define the CD127^+^ NK lineage in wild-type mice—specifically those with the phenotype integrin αv^+^Mac-1^−^, integrin αv^+^CD27^+^, integrin αv^+^CD11c^+^, and integrin αv^+^Ly49D^−^—were selectively absent, consistent with the loss of the CD127^+^ subset, while conventional NK cells (integrin αv^low^Mac-1^high^) developed normally (Figure 7b). In contrast, CD127^−^ NK cells displayed no significant differences in these markers compared with wild-type controls (Figure 7b), indicating that the absence of surrogate populations in IL-7Rα-deficient mice is not merely a consequence of the inability to detect the CD127 receptor protein; rather, it reflects a specific failure of this distinct lineage to develop or persist in the absence of functional IL-7 receptor signaling.

Collectively, these findings demonstrate that IL-7Rα signaling is essential for the development and maintenance of the subset that expresses CD127 in wild-type mice. Moreover, the preserved development of CD127^−^ NK cells in IL-7Rα^−^/^−^ mice indicates that CD127^+^ NK cells do not represent an immature precursor stage of CD127^−^ NK cells but instead constitute a developmentally distinct NK cell subset.

### 2.4. Murine CD127^+^ NK Cells Represent a Functionally Distinct NK Cell Subset

**Figure 8:**
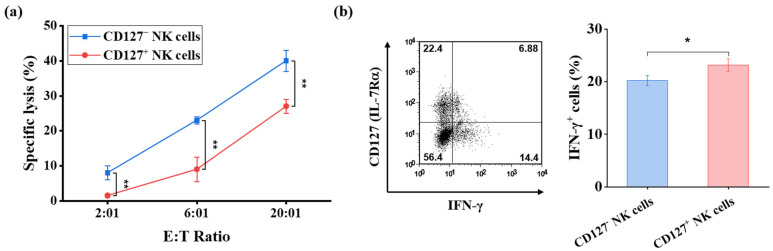
Functional characterization of murine CD127^+^ NK cells. (**a**) Cytotoxic activity of sorted, freshly isolated CD127^+^ and CD127^−^ NK cells from the spleen and lymph nodes of adult C57BL/6 mice (*n* = 5) was assessed against YAC-1 target cells using a ^51^Cr-release assay at the indicated effector-to-target (E/T) ratios. CD127^+^ NK cells exhibited measurable cytotoxic activity; however, their lytic capacity was significantly lower than that of CD127^−^ NK cells at multiple E/T ratios. (**b**) Intracellular IFN-γ production by CD127^+^ and CD127^−^ NK cells following stimulation with anti-NK1.1 mAb (PK136). Representative flow cytometric plots are shown, with numbers in the quadrants indicating the percentage of cells in each region. Data are presented as mean ± SD. Statistical comparisons between CD127^+^ and CD127^−^ subsets were performed using an unpaired two-tailed Student’s *t*-test. * *p* < 0.05, ** *p* < 0.01. IFN-γ—interferon-γ; mAb—monoclonal antibody.

To assess the functional capacity of CD127^+^ NK cells compared with that of CD127^−^ NK cells, NK cells were enriched from the spleen and lymph nodes of adult C57BL/6 mice using magnetic negative selection, followed by flow cytometric sorting of the respective subsets. Cytotoxic activity was assessed using a standard ^51^Cr-release assay against the NK-sensitive YAC-1 target cell line. Freshly isolated CD127^+^ NK cells exhibited measurable cytotoxic activity across multiple effector-to-target (E/T) ratios. However, their lytic activity was significantly lower than that of CD127^−^ NK cells (*p* < 0.01 where indicated; Figure 8a). Thus, although CD127^+^ NK cells display reduced expression of maturation-associated markers such as Mac-1, they retain cytotoxic function, albeit at a diminished level.

To further assess effector function, intracellular interferon (IFN)-γ production was analyzed following stimulation with anti-NK1.1 monoclonal antibody (PK136). CD127^+^ NK cells produced robust levels of IFN-γ (23.15 ± 1.21%, Figure 8b); as shown in Figure 8b, the frequency of IFN-γ-producing cells was significantly higher than that observed in the CD127^−^ NK cells (20.17 ± 0.98%, *p* < 0.01).

Collectively, these results demonstrate that CD127^+^ NK cells are functionally competent. Despite exhibiting reduced cytotoxic activity relative to CD127^−^ NK cells, they maintain—and slightly enhance—cytokine-producing capacity. These findings indicate that CD127^+^ NK cells represent a phenotypically and functionally distinct NK cell subset rather than a developmentally impaired population.

### 2.5. Murine CD127^+^ NK Cell Development Is Independent of IL-15 and IL-15Rα Signaling, in Contrast to CD127^−^ NK Cells

Previous studies have demonstrated that NK cells are virtually absent in IL-2Rβ^−/−^, IL-15^−/−^, and IL-15Rα^−/−^ mice, underscoring the essential role of IL-15 receptor signaling in conventional NK cell development [25,26,28]. To determine whether CD127^+^ and CD127^−^ NK cell subsets differ in their dependence on IL-15 and IL-15Rα signaling, we performed flow cytometric analyses of mononuclear cells isolated from the spleen, bone marrow, lymph nodes, liver, and thymus of IL-15^−/−^ and IL-15Rα^−/−^ mice.

**Figure 9:**
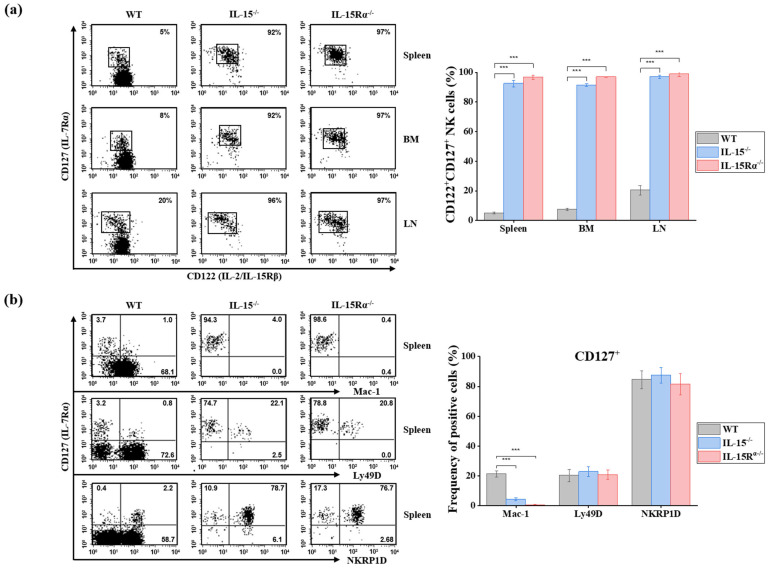
Murine CD127^+^ NK cell development is independent of IL-15 and IL-15Rα signaling, unlike CD127^−^ NK cells. (**a**) Representative flow cytometric analysis of CD127^+^ and CD127^−^ NK cells in the spleen, bone marrow (BM), and lymph nodes (LN) of wild-type (WT), IL-15^−/−^, and IL-15Rα^−/−^ mice (*n* = 3). Although total NK cell frequencies were markedly reduced in both knockout strains, CD127^+^ NK cells remained detectable, whereas CD127^−^ NK cells were virtually absent. Bar graphs summarize the proportion of CD127^+^ cells among gated NK cells. (**b**) Phenotypic analysis of splenic CD127^+^ NK cells assessing surface expression of Mac-1, Ly49D, and NKRP1D. No significant differences in Ly49D or NKRP1D expression were observed among WT, IL-15^−/−^, and IL-15Rα^−/−^ mice. In contrast, Mac-1 expression was significantly reduced in CD127^+^ NK cells from IL-15^−/−^ and IL-15Rα^−/−^ mice compared with WT controls. Numbers in quadrants indicate the percentage of cells within each region. Data are presented as mean ± SD. Statistical significance was determined by one-way ANOVA followed by Dunnett’s multiple comparisons test and is indicated as *** *p* < 0.001. IL—interleukin.

As expected, total NK cell frequencies were markedly reduced in both knockout strains compared with wild-type C57BL/6 controls. In the spleen, NK cell frequency within the lymphocyte gate was reduced by 96.8% in IL-15Rα^−/−^ mice and 96.1% in IL-15^−/−^ mice, while the proportion of NK cells decreased by 99.6% in γc^−/−^ mice, compared with wild-type controls (Figure 9a). Despite this profound reduction in NK cell population, CD127^+^ NK cells remained detectable in all examined tissues. Notably, the proportion of CD127^+^ cells among residual NK cells was dramatically increased in the absence of IL-15 signaling. In IL-15Rα^−/−^ mice, CD127^+^ NK cells comprised approximately 97% of NK cells in the spleen, bone marrow, and lymph nodes (*p* < 0.001, Figure 9a). Similarly, in IL-15^−/−^ mice, CD127^+^ NK cells accounted for approximately 92% in the spleen and BM and 96% in the lymph nodes (*p* < 0.001, Figure 9a). In contrast, CD127^−^ NK cells were nearly absent in these tissues in both knockout strains, indicating that CD127^−^ NK cell development is critically dependent on IL-15/IL-15Rα signaling, whereas CD127^+^ NK cells can arise independently of this pathway (Figure 9a).

To determine whether CD127^+^ NK cells developing in IL-15-deficient conditions retained their phenotypic characteristics, we examined surface expression of Mac-1, Ly49D, and NKRP1D in splenic CD127^+^ NK cells (Figure 9b). While the frequencies of Ly49D and NKRP1D expression were statistically comparable among strains (*p* > 0.05), a significant reduction in Mac-1 expression was observed in CD127^+^ NK cells from both knockout strains (4.2 ± 0.9% in IL-15^−/−^ mice and 0.5 ± 0.3% in IL-15Rα^−/−^ mice vs. 21.3 ± 2.2% in wild-type mice, all *p* < 0.001, Figure 9b).

These findings demonstrate that murine CD127^+^ NK cells develop independently of IL-15 and IL-15Rα signaling. Nevertheless, IL-15 may contribute to their optimal maturation or expansion, as reflected by reduced Mac-1 expression under IL-15-deficient conditions. Collectively, these data distinguish CD127^+^ NK cells from conventional CD127^−^ NK cells, which are critically dependent on IL-15 signaling for their development.

### 2.6. IL-7 Specifically Selectively Promotes Proliferation of Murine CD127^+^ NK Cells In Vitro and In Vivo

To determine whether CD127^+^ NK cells are functionally responsive to IL-7, we assessed proliferative responses of sorted CD127^+^ and CD127^−^ NK cells under various cytokine conditions.

**Figure 10:**
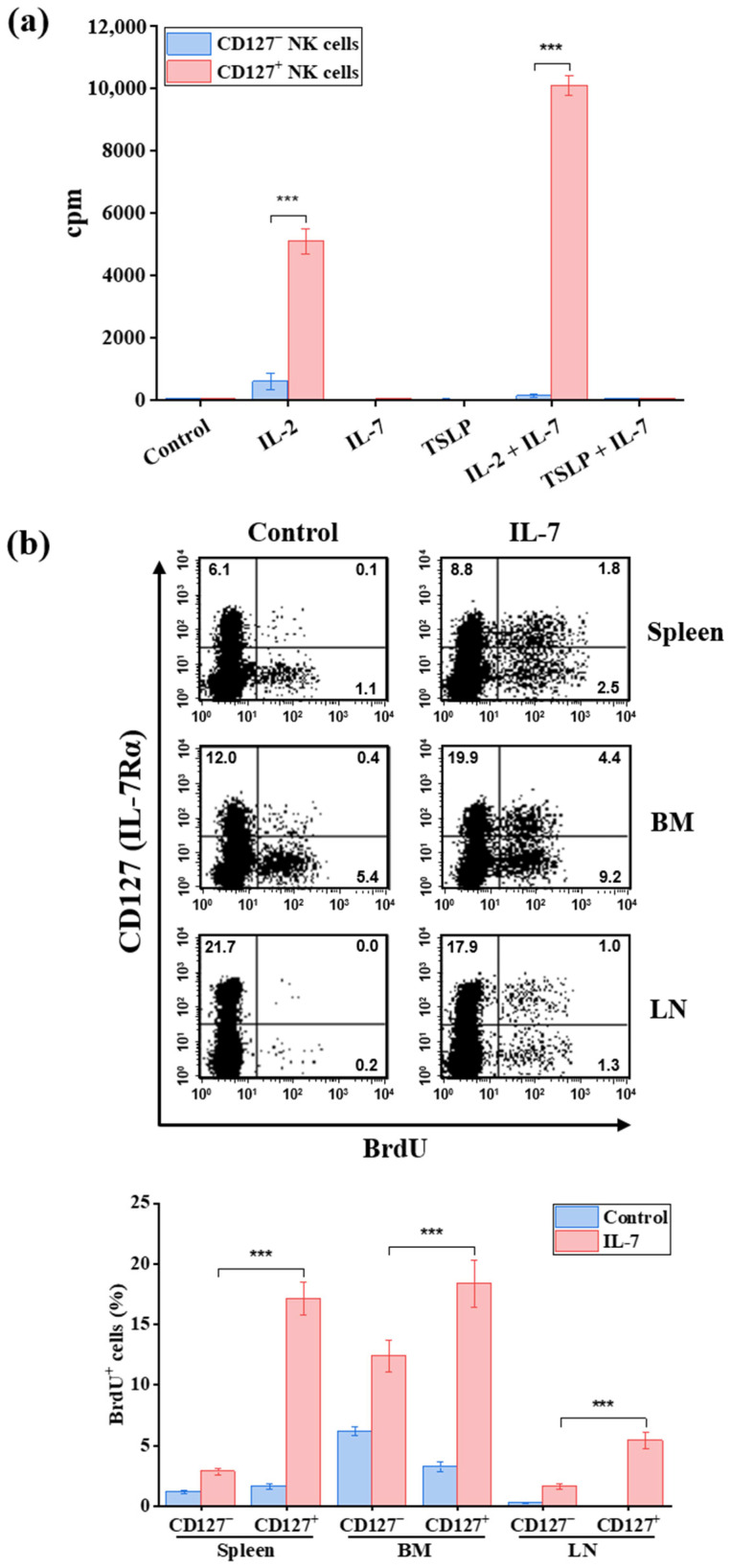
IL-7 selectively promotes proliferation of murine CD127^+^ NK cells in vitro and in vivo. (**a**) Proliferation of sorted CD127^+^ and CD127^−^ NK cell subsets was assessed by ^3^H-thymidine incorporation following in vitro stimulation with IL-2 (50 U/mL), IL-7 (100 ng/mL), TSLP (100 ng/mL), IL-2 plus IL-7, or TSLP plus IL-7 (*n* = 5). IL-7 significantly enhanced the proliferation of CD127^+^ NK cells, particularly in combination with IL-2, whereas minimal effects were observed in CD127^−^ NK cells. Bar graphs represent proliferation as counts per minute (CPM). (**b**) In vivo proliferation was assessed by BrdU incorporation in CD127^+^ and CD127^−^ NK cells from the spleen, bone marrow (BM), and lymph nodes (LN) of C57BL/6 mice treated with recombinant murine IL-7 (20 µg/mouse/day for three consecutive days; *n* = 3). Data are expressed as the percentage of BrdU^+^ cells within each subset. Representative flow cytometric plots are shown, with numbers in the quadrants indicating the percentage of cells in each region. Data are presented as mean ± SD. Statistical significance was determined using an unpaired two-tailed Student’s *t*-test and is indicated as *** *p* < 0.001. IL—interleukin; TSLP—thymic stromal lymphopoietin.

In vitro, cell proliferation was measured by ^3^H-thymidine incorporation following stimulation with IL-2, IL-7, thymic stromal lymphopoietin (TSLP), IL-2 plus IL-7, or TSLP plus IL-7 (Figure 10a). IL-7 alone did not significantly induce proliferation in either subset. However, IL-7 markedly enhanced the proliferation of CD127^+^ NK cells when combined with a low concentration of IL-2, demonstrating a clear synergistic effect (Figure 10a). Proliferation of CD127^+^ NK cells following IL-2 plus IL-7 stimulation was significantly higher than that of CD127^−^ NK cells (approximately 68.7-fold; *p* < 0.001; Figure 10a). In contrast, IL-7 did not confer a comparable additive effect on CD127^−^ NK cells under the same conditions (Figure 10a).

Consistent with this selective responsiveness, CD127^+^ NK cells exhibited substantially greater proliferative responses to IL-2-based stimulation compared with CD127^−^ NK cells (*p* < 0.001, Figure 10a). Stimulation with TSLP, either alone or in combination with IL-7, failed to induce proliferation in either subset, despite sharing the IL-7Rα chain (Figure 10a).

To evaluate proliferative responses in vivo, C57BL/6 mice were treated with recombinant IL-7 (20 µg/mouse/day for three consecutive days), and BrdU incorporation was assessed in splenic, bone marrow, and lymph node NK cells (Figure 10b). IL-7 administration significantly increased BrdU incorporation in CD127^+^ NK cells compared with CD127^−^ NK cells across all examined tissues (Figure 10b). The relative increase in proliferation of CD127^+^ NK cells was approximately 6.0-fold in the spleen, 1.5-fold in the BM, and 3.3-fold in the LN (all *p* < 0.001).

Collectively, these results demonstrate that murine CD127^+^ NK cells are selectively responsive to IL-7 signaling both in vitro and in vivo. This proliferative responsiveness further distinguishes CD127^+^ NK cells from CD127^−^ NK cells and supports the conclusion that CD127^+^ NK cells represent an IL-7-dependent NK cell subset.

## 3. Discussion

Despite the well-established division of human NK cells into CD56^dim^ and CD56^bright^ subsets, a parallel classification for murine NK cells remains unresolved [47,48,61,62,63,64,65,66,67]. A previous study identified a population of CD127^+^ NK cells in mice with the phenotype DX5^+^CD127^+^NK1.1^+^CD122^+^CD3^−^CD19^−^ and proposed that these cells are thymus-derived and functionally analogous to human CD56^bright^ NK cells, whereas CD127^−^ NK cells originate from the bone marrow and resemble human CD56^dim^ NK cells [51]. In this study, we provide a comprehensive characterization of CD127^+^ NK cells across multiple organs, revealing key discrepancies with this earlier report that challenge the model of a solely thymic origin.

One major discrepancy lies in the reported frequency of CD127^+^ NK cells in the thymus of C57BL/6 mice. The previous study, which primarily examined CD127^+^ NK cells within the thymus, reported that approximately 85% of thymic NK cells were CD127^+^ [51]. In contrast, our study found this population represented only ~20% of gated NK cells. Interestingly, in B6.RAG1^−/−^ mice, which lack mature T and B cells, the frequency of thymic CD127^+^ NK cells increased to ~80%, aligning with the previous report and suggesting that the presence of other lymphoid cells may influence this measurement [51].

More critically, our evaluation of athymic nude (Foxn1^−/−^) mice demonstrated that the frequencies of CD127^+^ NK cells in the spleen, bone marrow, lymph nodes, and liver were comparable with wild-type controls, directly contradicting the previous report of their marked reduction [51]. This observation suggests that while the thymus can generate CD127^+^ NK cells, a substantial thymus-independent lineage exists that populates peripheral tissues even in the absence of a thymus. This challenges the hypothesis that the thymus is the exclusive source of CD127^+^ NK cells in peripheral tissues. While our data from athymic (Foxn1^−/−^) mice compellingly demonstrate that the majority of peripheral CD127^+^ NK cells develop via a thymus-independent pathway, we acknowledge that these results do not exclude the possibility of a minor thymic contribution in wild-type settings. It is plausible that thymus-derived emigrants contribute to the peripheral CD127^+^ pool, a phenomenon that may be particularly relevant for populations localized within the lymph nodes of wild-type mice. These discrepancies likely stem from methodological differences, including broader gating strategies in our study (CD122^+^CD3^−^CD19^−^NK1.1^+^), in contrast to their restricted gating to DX5^+^CD122^+^CD3^−^CD19^−^NK1.1^+^ cells [51].

Phenotypic analysis of murine CD127^+^ NK cells revealed that these Mac-1^low^CD43^low^DX5^low^Ly49^neg/low^ cells resemble NK cells at an intermediate stage of bone marrow development [8,9]. This raised the possibility that they are an immature precursor population. To test this hypothesis, we examined their status in fetal and NKD mice. If CD127^+^ NK cells were early-stage precursors, they would be expected to predominate in these models; however, their frequency and phenotype remained comparable to those in adult mice, challenging their classification as transient immature precursors.

This conclusion is bolstered by findings in IL-7Rα^−/−^ mice, which exhibit a selective, complete loss of CD127^+^ NK cells, while the CD127^−^ population remains entirely intact. If CD127^+^ NK cells were precursors to CD127^−^ NK cells, their loss would substantially impact the latter population, which was not observed. This selective loss confirms that CD127^+^ NK cells represent a distinct lineage dependent on IL-7 signaling, not a transient precursor stage of CD127^−^ NK cells.

Functional analyses solidified their status as a mature population. CD127^+^ NK cells from the spleen and lymph nodes retain substantial killing capacity—albeit lower than their CD127^−^ counterparts—and effectively produce IFN-γ. This functional competence reinforces their parallel to the human CD56^bright^ NK cell subset, which is also characterized by high cytokine production, lower cytotoxicity, and expression of CD127 [46,68]. This inverse relationship between cytokine production and cytotoxicity suggests a functional specialization, although our data do not establish a direct causal link.

Additionally, this functional attribute is a major point of divergence from ILC1s; while both populations produce robust levels of IFN-γ upon stimulation, ILC1s are primarily cytokine-producing “helper” cells and generally lack the potent lytic activity characteristic of the NK cell lineage [52,53,54,55,56]. The maintenance of substantial cytotoxicity in our CD127^+^ subset, coupled with the presence of maturation-associated markers, confirms their identity as mature NK cells rather than helper ILC1s. Crucially, while CD127 expression is a defining feature of helper-like ILCs, our findings distinguish the CD127^+^ population from ILC1s based on phenotypic and functional criteria. Unlike ILC1s, which are typically defined by a CD127^+^DX5^−^Mac-1^−^ phenotype [52,53,54,55,56], the cells identified here maintain significant expression of terminal maturation markers, including Mac-1 and DX5, as well as substantial cytotoxic activity. While these markers and functional data strongly support an NK cell identity, future inclusion of Eomesodermin (Eomes) expression would further clarify the precise lineage boundaries between these CD127^+^ NK cells and ILCs [52,53,54,55,56].

It is plausible that the distinct cytokine dependencies we identified—IL-7 for CD127^+^ and IL-15 for CD127^−^ NK cells—are instrumental in establishing and maintaining these specialized functional profiles. The observed inverse relationship between cytokine production and cytotoxicity in the CD127^+^ subset is consistent with the hallmark features of human CD56^brigh^ NK cells, which exhibit reduced cytotoxicity but enhanced cytokine production. Together, these findings demonstrate that CD127^+^ NK cells are functionally competent and constitute a mature NK cell population with distinct effector characteristics. This analogy is strengthened by the enrichment of CD127^+^ NK cells in lymph nodes, mirroring the preferential localization of human CD56^bright^ NK cells [46,69,70]. However, while murine CD127^+^ NK cells share phenotypic and functional similarities with human CD56^bright^ NK cells, it remains premature to consider them direct equivalents without further investigation.

A fundamental finding of this study is the distinct cytokine dependency that separates these two lineages. IL-15 is classically considered essential for NK cell development and peripheral survival. Indeed, mature CD127^−^ NK cells fail to persist in IL-15-deficient hosts. Remarkably, CD127^+^ NK cells continue to develop in IL-15^−/−^ and IL-15Rα^−/−^ mice, displaying normal surface marker profiles despite reduced absolute numbers. This indicates that murine CD127^+^ NK cells can develop independently of IL-15 signaling, in contrast to CD127^−^ NK cells, which are strongly IL-15-dependent, although they exhibit altered phenotypic profiles characterized by a significant failure to upregulate Mac-1, suggesting that IL-15 signaling is critical for the terminal maturation stages of this subset. These results significantly revise the established model, where the absence of IL-15 leads to a near-complete loss of peripheral NK cells. The residual NK cells observed in previous studies using IL-15 blockade or IL-15^−/−^ and IL-15Rα^−/−^ mice may, in fact, correspond to this IL-15-independent CD127^+^ subset [28,30,71,72].

In contrast to their IL-15 independence, CD127^+^ NK cells are strictly dependent on IL-7 for their development and homeostasis. We demonstrated that IL-7Rα deficiency selectively impairs the development of CD127^+^ NK cells without affecting CD127^−^ populations and robustly stimulates their proliferation both in vitro and in vivo. While our data clearly show that IL-7 signaling drives proliferation, its potential role in promoting survival also warrants discussion. In other lymphoid lineages, particularly T cells, IL-7 is a critical survival factor that functions by upregulating anti-apoptotic proteins such as Bcl-2 and Mcl-1 [73]. Given that IL-7Rα deficiency leads to a complete loss of the CD127^+^ NK cell subset, it is highly plausible that IL-7 provides essential survival signals in addition to its proliferative effects. This dual function would ensure the maintenance and homeostatic balance of this distinct NK cell population. Future studies investigating the expression of anti-apoptotic molecules in CD127^+^ NK cells following IL-7 stimulation would be valuable to formally dissect the contributions of proliferation versus survival. These findings challenge the long-held consensus that IL-7 is dispensable for NK cell development and biology—a perspective shaped largely by studies conducted before the recognition of the CD127^+^ NK cell subset [74,75,76,77,78,79].

In conclusion, this work identified a mature, IL-7-dependent, IL-15-independent, and largely thymus-independent CD127^+^ NK cell population. Based on the phenotype, tissue distribution, and functional properties, the mature murine NK cell compartment comprises at least two distinct lineages governed by separate regulatory mechanisms: CD127^−^ NK cells, which resemble human CD56^dim/neg^ NK cells, and CD127^+^ NK cells, which share several features with the human CD56^bright^ subset.

Future studies are warranted to elucidate the developmental origins, lineage relationships, and specific immunological roles of CD127^+^ NK cells. A deeper investigation into their effector capabilities, including their capacity for chemokine production, and their potential unique immunoregulatory functions or tissue-specific responses in contexts such as infection, autoimmunity, and cancer, will be critical for fully understanding their contribution to the innate immune system.

## 4. Materials and Methods

### 4.1. Mice

C57BL/6 (B6), B6.RAG-1^−/−^ (B6.129S7-Rag1^tm1Mom^/J), B6.β2m^−/−^ (C57BL/6J-B2m^tm1Unc^), nude (B6.Cg-Foxn1^nu^; C57BL/6J-Hfh11^nu^), B6.IL-2Rγ^−/−^ (B6.129S4-Il2rg^tm1Wjl^/J), B6.IL-7Rα^−/−^ (B6.129S7-Il7r^tm1Imx^/J; Il7r^tm1Imx^), and B6.IL-15Rα^−/−^ (B6;129X1-Il15ra^tm1Ama^/J; B6;Il15ra^tm1Ama^) mice were obtained from The Jackson Laboratory (Bar Harbor, ME, USA). Additional C57BL/6 mice were obtained from the National Cancer Institute (Frederick, MD, USA). B6.IL-15^−/−^ (C57BL/6NTac-Il15^tm1Imx^) mice were purchased from Taconic Biosciences (Germantown, NY, USA).

The NKD transgenic mouse model, characterized by selective NK cell deficiency owing to a transgene insertion in the activating transcription factor 2 (ATF2) locus, a member of the basic leucine zipper (bZIP) transcription factor family, exhibits substantially reduced peripheral NK cell numbers and functional immaturity [80,81]. In NKD mice, NK cell development appears to arrest after acquisition of the pan-NK marker DX5 and downregulation of αvβ3 integrin, occurring prior to the upregulation of Mac-1 and CD43, which leads to accumulation of immature NK cells in the bone marrow. Although the precise mechanisms underlying this immature phenotype are under investigation [82], NKD mice have been instrumental in elucidating aspects of NK cell development.

All mice were housed under specific pathogen-free conditions and used at 8–14 weeks of age unless otherwise indicated. The experimental work was conducted by the corresponding author in accordance with institutional animal care guidelines during a prior research stay abroad, before the preparation of this manuscript.

### 4.2. Antibodies and Reagents

The following fluorochrome-conjugated mAbs and streptavidin-conjugates were purchased from BD Biosciences (San Jose, CA, USA): Phycoerythrin (PE)- or allophycocyanin (APC)-conjugated anti-NK1.1 (PK136); fluorescein isothiocyanate (FITC)- or PE-conjugated anti-IL-2Rβ (CD122, clones 5H4 or TM-β1); PerCP-Cy5.5-conjugated anti-CD3e (145-2C11); FITC- or PE-anti-CD4 (RM4-5); FITC- or PE-anti-CD8a (53-6.7); PE-Cy5-conjugated anti-TCRβ (H57-597); FITC- or PE-anti-CD11b (Mac-1; M1/70); FITC- or PE-anti-CD11c (HL3); PerCP-Cy5.5-conjugated anti-CD19 (1D3); PE- or biotin-anti-CD27 (LG.3A10); FITC-anti-CD43 (Ly-48; S7); FITC-anti-CD49b (DX5), PE- or biotin-anti-CD51 (integrin αv; H9.2B8); PE- or biotin-anti-CD61 (integrin β3, 2C9.G2); FITC- or PE-anti-c-Kit (CD117; 2B8); FITC- or PE-anti-Ly-49A (JR9-318 or A1); PE-anti-Ly49F (HBF-719); FITC- or PE-anti-Ly-49G2 (LGL-1; 4D11 or Cwy-3); FITC-anti-Ly-49I (YLI-90); PE-anti-Ly-49C/I (5E6); FITC- or biotin-anti-NKG2A/C/E (20d5); and FITC- PE- or APC- streptavidin.

FITC-anti-CD3e (145-2C11), FITC-CD27 (LG.7F9), PE- or biotin-anti-CD127 (A7R34), PE−anti-Ly49A/D (12A8), PE-anti-Ly49-49C/I/F/H (14B11), PE-anti-NKG2A/C/E (20d5), PE-anti-NKG2A (16a11), and PE-anti-NKG2D (A10 or CX5) were procured from e-Bioscience (San Diego, CA, USA). Alexa Fluor 488–streptavidin was obtained from Invitrogen (Carlsbad, CA, USA).

The following mAbs were purified in the Yokoyama laboratory at Washington University and conjugated to FITC or biotin using standard protocols: anti-Ly49A (JR9-318, a gift from J. Roland); anti-Ly49D (4E4) [83]; anti-Ly49G2 (4D11) [84]; anti-Ly49H (3D10) [85]; biotin-anti-KLRG1; and biotin-anti-NKRP1D (2D12) [86]. The 2.4G2 hybridoma (anti-FcγRII/III) was obtained from the American Type Culture Collection (ATCC; Manassas, VA, USA). Recombinant murine IL-7 and IL-15 were purchased from PeproTech (Rocky Hill, NJ, USA), and recombinant mouse TSLP was obtained from R&D Systems (Minneapolis, MN, USA).

### 4.3. Cell Preparation and Flow Cytometry

Immunophenotypic analyses were performed using four-color flow cytometry on a FACSCalibur (BD Biosciences) equipped with CellQuest software (version 5.2, BD Biosciences). Single-cell suspensions of the spleen, bone marrow, lymph nodes, liver, and thymus were prepared using standard mechanical dissociation techniques and depleted of red blood cells [87]. The number of viable cells isolated from each tissue was determined by counting live cells using trypan blue exclusion.

For surface staining, cells were first incubated with 2.4G2 anti-FcγRII/III antibody (Fc receptor blocking reagent) to prevent nonspecific antibody binding. Cells were subsequently stained with combinations of fluorochrome-conjugated or biotinylated mAbs as indicated. When biotinylated mAbs were used, cells were subsequently incubated with FITC-, PE-, or APC-conjugated streptavidin. Background fluorescence was assessed using fluorochrome-labeled, isotype-matched nonreactive control mAbs.

**Figure 11:**
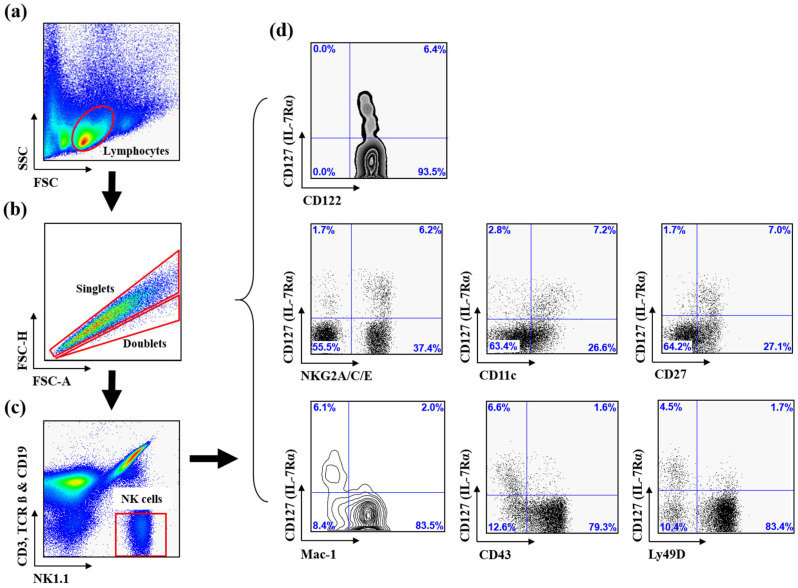
Gating strategy for the identification of murine CD127^+^ and CD127^−^ NK cell subsets. Sequential flow cytometric gating strategy used to identify and characterize murine NK cells from single-cell suspensions of spleen tissue. (**a**) Lymphocyte gate: Representative flow cytometry plots illustrating the initial electronic gate set on the small lymphocyte population based on forward scatter (FSC) and side scatter (SSC) characteristics following red blood cell lysis and Fc receptor blocking with 2.4G2 antibody. (**b**) Singlet discrimination: Doublets were excluded by FSC-A versus FSC-H gating to ensure analysis of single cells. (**c**) NK cell identification: Within the lymphocyte gate, NK cells were identified as NK1.1^+^ cells negative for T cell markers (CD3, TCRβ) and B cell markers (CD19), yielding a purified NK cell population defined as NK1.1^+^CD3^−^TCRβ^−^CD19^−^ cells. CD122 expression was consistently detected within this gate, further confirming NK cell identity. (**d**) Subset characterization: Representative flow cytometric plots showing differential expression of CD127 and additional maturation and receptor markers, such as Mac-1, CD43, Ly49D, NKG2A/C/E, CD27, and CD11c, within the CD127^+^ and CD127^−^ NK cell subsets. Numbers in quadrants indicate the percentage of cells within each region.

Flow cytometric analysis was performed using a sequential gating strategy (Figure 11). As illustrated in Figure 11, lymphocytes were first gated based on forward scatter (FSC) and side scatter (SSC) characteristics. Doublets were excluded by FSC-A versus FSC-H gating to ensure analysis of singlet events. Within this gate, NK cells were identified as NK1.1^+^ cells negative for lineage markers CD3, TCRβ, and CD19 (NK1.1^+^CD3^−^TCRβ^−^CD19^−^). CD122 expression was consistently detected within this population, further confirming accurate NK cell identification. Subsequent analyses were performed to distinguish CD127^+^ and CD127^−^ NK cell subsets and to assess surface expression of maturation- and receptor-associated NK cell markers.

### 4.4. Induction of IFN-γ Production and Intracellular IFN-γ Staining

For in vitro stimulation of NK cells, single-cell suspensions prepared from the spleen—and in some cases, lymph nodes—were incubated with plate-bound anti-NK1.1 mAb (PK136; BD Biosciences) for 30 min. Subsequently, cells were further incubated in the presence of brefeldin A for an additional 6–8 h to block cytokine secretion.

For intracellular IFN-γ staining, cells were first stained for surface markers, then fixed and permeabilized using the Cytofix/Cytoperm kit (BD Biosciences), followed by staining with FITC- or Alexa Fluor 488-conjugated anti-IFN-γ mAb (XMG1.2; BD Biosciences), according to the manufacturer’s instructions.

### 4.5. Purification of CD127^+^ and CD127^−^ NK Cell Subsets

Single-cell suspensions from the spleen and lymph nodes were prepared using standard techniques. Mouse NK cells were isolated by depleting non-NK cells from the leukocyte population using a MACS NK cell isolation kit (negative selection), according to the manufacturer’s instructions (Miltenyi Biotec, Bergisch Gladbach, Germany).

Briefly, non-NK cells were indirectly magnetically labeled using a cocktail of biotin-conjugated mAbs against CD4 (L3T4), CD8a (Ly-2), CD5 (Ly-1), CD19, Ly-6G (Gr-1), and Ter-119, followed by anti-biotin microbead-conjugated antibodies. The labeled non-NK cells were retained on a MACS column in a magnetic field, while unlabeled NK cells were collected in the flow-through.

Thereafter, NK cells were stained with FITC-anti-CD3, PE-anti-CD127, APC-anti-NK1.1, and corresponding isotype control antibodies, and sorted using a MoFlo high-performance cell sorter (DakoCytomation, Glostrup, Denmark) to purify CD127^+^ and CD127^−^ NK cell subsets. The purity of the CD127^−^ NK cells typically exceeded 99%, and that of the CD127^+^ NK cells exceeded 95%, as assessed by flow cytometry.

### 4.6. In Vitro Proliferation Assays

Thymidine incorporation assays were performed using sorted CD127^+^ and CD127^−^ mouse NK cell subsets, which were plated in triplicate in 96-well plates. Cells were incubated in R10 medium, consisting of RPMI 1640 (Sigma-Aldrich, St. Louis, MO, USA) supplemented with 10% fetal calf serum (FCS; HyClone, Logan, UT, USA), 2-mercaptoethanol (Sigma-Aldrich), L-glutamine (Sigma-Aldrich), penicillin (Sigma-Aldrich), and streptomycin (Sigma-Aldrich), in the presence of IL-2 (50 U/mL), IL-7 (100 ng/mL), or TSLP (100 ng/mL).

After 48 h of incubation, ^3^H-thymidine (0.4 μCi/well; PerkinElmer, Waltham, MA, USA) was added for the final 24 h. At 72 h, plates were washed, and cells were harvested onto filter mats (Tomtec, Hamden, CT, USA). Incorporated ^3^H-thymidine was measured using a liquid scintillation counter (Wallac, Gaithersburg, MD, USA) and recorded as counts per min (cpm) per well.

### 4.7. Cytotoxicity Assay

To compare the cytotoxic function of CD127^+^ and CD127^−^ NK cell subsets, cells from the spleen and lymph nodes of C57BL/6 mice were sorted, as previously described. The sorted subsets were directly tested using a standard ^51^Cr-release assay.

Briefly, NK cell-mediated cytotoxicity was assessed by measuring ^51^Cr-release following the incubation of freshly isolated, unstimulated effector cells with ^51^Cr-labeled YAC-1 target cells (ATCC). Using 96-well V-bottom plates, target cells were incubated with effector cells in triplicate and a total volume of 200 µL at various effector-to-target (E/T) ratios for 4 h. Thereafter, the plates were centrifuged prior to incubation, and after 4 h, supernatants were harvested, and radioactivity was measured using a gamma counter.

### 4.8. Detection of Proliferating Cells In Vivo After IL-7 Treatment

Adult C57BL/6 mice were intraperitoneally administered with 20 µg of recombinant murine IL-7 (Peprotech) daily for 3 or 4 consecutive days. Mice received an intraperitoneal injection of 2 mg of BrdU (BrdU Flow Kit, BD Biosciences) 24 h after the final IL-7 injection. At least two untreated mice were included in each experiment as BrdU-negative controls.

Single-cell suspensions were prepared from various tissues in cold phosphate-buffered saline, supplemented with 1% FCS and 0.09% sodium azide 3 h after BrdU administration. Red blood cells were lysed, and cells were incubated with 2.4G2 culture supernatant to block nonspecific Fc receptor binding.

Thereafter, the cells were surface-stained with the following antibodies: PE- or biotin-conjugated anti-CD127, PerCP-Cy5.5 anti-CD3ε, PerCP-Cy5.5 anti-CD19, PE-Cy5 anti-TCRβ, and APC anti-NK1.1. When biotinylated antibodies were used, cells were further incubated with PE-streptavidin. For BrdU staining, cells were fixed, permeabilized, treated with DNase, and then incubated with FITC-anti-BrdU, according to the manufacturer’s instructions (BD Biosciences BrdU Flow Kit).

### 4.9. Statistical Analysis

All quantitative results are expressed as mean ± standard deviation (SD) from at least three independent experiments. Comparisons between two groups were performed using Student’s *t*-test. Values of * *p* < 0.05, ** *p* < 0.01, and *** *p* < 0.001 were considered statistically significant.

## 5. Conclusions

In conclusion, although the classification of murine NK cell subsets remains incomplete, the identification of peripheral CD127^+^ NK cells as a novel, mature, and largely thymus-independent population provides a new framework for understanding NK cell-mediated immunity. This subset displays distinct phenotypic and functional characteristics—including substantial cytotoxic capacity and robust IFN-γ production—that distinguish them from helper-like ILC1s and suggest an alternative developmental pathway governed by selective IL-7 responsiveness. These findings highlight the complexity of NK cell ontogeny and suggest the existence of previously unrecognized regulatory mechanisms governing NK cell diversity. Ultimately, this work expands our current understanding of NK cell biology and lays the groundwork for future translational research in fields such as vaccination, infection, inflammation, autoimmunity, and cancer.

## Data Availability

The original contributions presented in this study are included in the article. Further inquiries can be directed to the corresponding author upon request.

## Abbreviations

ATF2	Activating transcription factor 2
Bach2	BTB and CNC homology 1, basic leucine zipper transcription factor 2
Bcl-2	B-cell lymphoma-2
Bim	Bcl-2-interacting mediator of cell death
β2m	β2-microglobulin
bZIP	Basic leucine zipper
CLP	Common lymphoid progenitors
Eomes	Eomesodermin
Foxn1	Forkhead box N1.
GATA-3	GATA binding protein 3
HSC	Hematopoietic stem cell
IL-7	Interleukin-7
IL-15	Interleukin-15
IFN-γ	Interferon-γ
JAK3	Janus kinase 3
KLRG1	Killer cell lectin-like receptor subfamily G member 1
mAb	Monoclonal antibody
Mac-1	Macrophage-1 antigen)
MACS	Magnetic activated cell sorting
Mcl-1	Myeloid cell leukemia 1
NK cells	Natural killer cells
NKD	NK cell-deficient
NKG2	NK group 2 family of receptors
NKRP1D	Natural killer cell receptor P1D
NKP	NK progenitor
Noxa	Phorbol-12-myristate-13-acetate-induced protein 1 (PMAIP1)
T-bet	T-box expressed in T cells
T/NKP	T/NK progenitors
TCRβ	T-cell receptor β,
TSLP	thymic stromal lymphopoietin
Zeb2	Zeb2, zinc finger E-box-binding homeobox 2
γc	The common γ chain (CD132)
